# Atmospheric Washout Dynamics of Organic Micropollutants: A Study of PAH, PAE, and BTEX Concentrations in Rainwater Across Northern Serbia

**DOI:** 10.3390/jox16030116

**Published:** 2026-06-20

**Authors:** Brankica Kartalović, Rastko Tomanović, Kristina Habschied, Alma Mikuška, Mirta Sudarić Bogojević, Antonije Žunić, Dora Bjedov

**Affiliations:** 1 BioSense Institute, University of Novi Sad, Dr Zorana Đinđića 1, 21000 Novi Sad, Serbia; brankica.kartalovic@biosens.uns.ac.rs; 2 Faculty of Sciences, University of Novi Sad, Trg Dositelja Obradovića 3, 21000 Novi Sad, Serbia; 3 Faculty of Food Technology Osijek, Josip Juraj Strossmayer University of Osijek, Franje Kuhača 18, 31000 Osijek, Croatia; 4 Department of Biology, Josip Juraj Strossmayer University of Osijek, Cara Hadrijana 8/A, 31000 Osijek, Croatia; 5 Faculty of Agriculture, University of Novi Sad, Trg Dositeja Obradovića 8, 21000 Novi Sad, Serbia

**Keywords:** rainwater, wet deposition, PAHs, phthalates, BTEX, source apportionment, GC-MS, atmospheric pollution, Vojvodina

## Abstract

Atmospheric wet deposition represents a major pathway for the transfer of organic micropollutants into terrestrial and aquatic ecosystems. This study investigates the occurrence and spatial distribution of polycyclic aromatic hydrocarbons (PAHs), phthalate esters (PAEs), and BTEX compounds in rainwater across Northern Serbia (Vojvodina region). Rainwater samples were collected during the 2025–2026 heating season at three locations: a petrochemical site in Kikinda, a traffic- and residentially influenced site in Sremska Mitrovica, and an urban background site in Sombor. Total concentrations showed pronounced spatial variability, with the highest ΣBTEX and ΣPAE levels recorded in Kikinda (∑BTEX = 2.818 μg L^∑1^; ∑PAE = 0.930 μg L^∑1^). Diagnostic ratios identified a dominant petrogenic signature in Kikinda (LMW/HMW > 1), while pyrogenic sources prevailed in Sremska Mitrovica and Sombor ((Fla/Fla + Pyr) > 0.5). BTEX profiles across all sites were characterised by the absence of benzene and elevated toluene and xylene levels (B/T ≈ 0; T/X > 1). Health risk assessment indicated an acceptable but non-negligible carcinogenic risk from PAHs, particularly for children in industrial areas. These findings highlight the role of precipitation as an efficient scavenger of organic pollutants and emphasise the need for integrated atmospheric–hydrological monitoring frameworks in industrialised regions.

## 1. Introduction

Atmospheric precipitation serves as a fundamental scavenging mechanism for the removal of gaseous and particle-bound pollutants from the troposphere. Through complex wet deposition processes, these pollutants are effectively transferred into terrestrial and aquatic ecosystems [1]. In industrialised regions such as Vojvodina (Northern Serbia), the atmosphere is impacted by a multi-component mixture of organic micropollutants. These include polycyclic aromatic hydrocarbons (PAHs), phthalate acid esters (PAEs), and volatile organic compounds (VOCs) such as benzene, toluene, ethylbenzene, and xylene isomers (BTEX). These compounds are continuously emitted from various anthropogenic sources, ranging from petrochemical operations and heavy traffic to residential biomass combustion and the degradation of plastic materials [2,3]. Recent monitoring within the Pannonian Basin has demonstrated that the regional atmospheric profile is not merely a product of local emissions. It is significantly influenced by long-range atmospheric transport (LRAT) from surrounding industrial clusters across Central and Southeastern Europe. Consequently, high-resolution spatial studies are necessary to disentangle transboundary signatures from localised pollution hotspots [4]. Among these pollutants, BTEX compounds are primary precursors for tropospheric ozone formation and secondary organic aerosols (SOA). While benzene is a Group 1 human carcinogen, its alkylated derivatives, such as toluene and xylenes, exhibit neurotoxic effects and contribute to the chemical load of receiving aquatic systems [5]. Semi-volatile PAHs and PAEs are also of concern because of their environmental stability, bioaccumulative potential, and endocrine-disrupting properties [6]. Although PAEs are not intentionally emitted into the atmosphere, their widespread use in plastics, packaging materials, construction products, and consumer goods results in continuous environmental release and atmospheric transport [3]. At present, neither Serbian nor European environmental legislation defines specific concentration limits for PAEs in rainwater or ambient air. Consequently, environmental assessments are generally based on occurrence monitoring, source identification, and risk evaluation approaches. Emerging research focuses on the synergistic relationship between these chemicals and microplastics. Phthalates, in particular, are recognised as molecular markers of plastic-mediated pollution, acting as transport-associated compounds that adsorb onto or leach from airborne microplastics [6]. The efficiency of wet scavenging is governed by a pollutant’s physicochemical properties, such as vapour pressure and Henry’s law constant, alongside dynamic meteorological conditions [7]. While volatile BTEX isomers are primarily removed through gas-phase diffusion, high-molecular-weight PAHs and PAEs are predominantly associated with aerosols. Their removal depends on the “washout” of particulate matter, which is sensitive to rainfall intensity and droplet size distribution [8].

Despite the growing body of literature on urban air quality, integrated studies that simultaneously quantify PAHs, PAEs, and BTEX in precipitation remain scarce. This lack of synchronised data creates a significant missing link in understanding the total pollutant flux entering soil and groundwater systems, which are critical for the agricultural sustainability of Northern Serbia. To address this complexity, this study applies a robust methodology driven by a combination of advanced analytical, chemometric, and risk-assessment tools. Dominant emission sources and transport pathways are identified using high-sensitivity GC-MS/MS in Selected Ion Monitoring (SIM) mode, coupled with advanced multivariate statistical tools, specifically Principal Component Analysis (PCA) and diagnostic ratio analysis, which serve as powerful tools for multi-component source apportionment. Furthermore, the toxicological significance of this synchronised dataset is evaluated through an integrated human health risk assessment, utilizing the standardized USEPA Incremental Lifetime Cancer Risk (ILCR) and Hazard Quotient (HQ) modelling tools to differentiate cumulative exposure between sensitive (children) and adult populations. This research aims to establish a baseline for integrated atmospheric-hydrological monitoring in industrialized Pannonian regions. To the best of our knowledge, this is the first study in the Pannonian Basin that integrates PAHs, PAEs, and BTEX in rainwater within a single framework combining these advanced source apportionment, flux estimation, and risk modelling tools.

## 2. Materials and Methods

### 2.1. Sampling Sites

**Figure 1:**
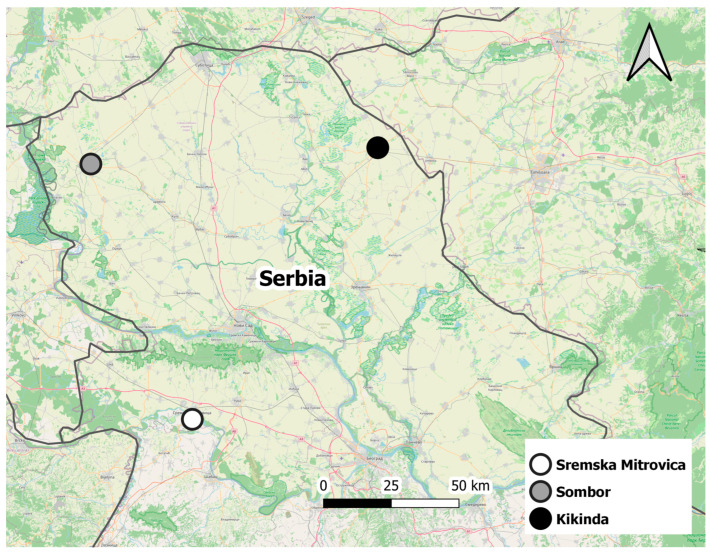
Sampling locations in Vojvodina (Northern Serbia): Sremska Mitrovica, Sombor and Kikinda.

Rainwater samples were collected between December 2025 and April 2026 at three sites in the Vojvodina province: Kikinda (*n* = 15), representing an industrial and petrochemical centre; Sombor (*n* = 13), acting as an urban background site; and Sremska Mitrovica (*n* = 12), characterized as a traffic and residential hub. Samples were collected using pre-cleaned stainless steel funnels connected to 1.5 L amber glass bottles. The sampling locations are illustrated in Figure 1.

### 2.2. Sampling Design

The sampling strategy followed a volume-weighted bulk approach rather than a discrete event-based protocol. Samples were retrieved once the collection vessel reached a cumulative volume of 1 L. This methodology was adopted to ensure a representative mass of organic micropollutants (PAHs, PAEs, and BTEX, Table 1) necessary for analytical detection, particularly in regions where individual rainfall events may be intermittent or of low intensity. To ensure Quality Assurance and Quality Control (QA/QC) during the sampling campaign and to prevent cross-contamination, strict protocols were implemented. All sampling was conducted using amber glass collectors and stainless-steel funnels to eliminate any potential leaching of target plasticizers (PAEs). Prior to deployment, all glassware and equipment were rigorously washed with phosphate-free detergent, rinsed sequentially with deionized water, acetone, and n-hexane, and finally baked at 400 °C for 4 h. Field blanks (consisting of pre-cleaned glass collectors filled with HPLC-grade water opened briefly during sample collection) and trip blanks were processed in parallel with each sampling batch to monitor potential ambient and transport-associated contamination. Target analytes in all field and trip blanks were either non-detectable or below the limit of quantification (LOQ). Immediately after collection, samples were sealed with Teflon-lined caps, wrapped in aluminium foil, transported to the laboratory in portable coolers at 4 °C, and stored in the dark at −20 °C until extraction to prevent photodegradation and volatilisation losses. By integrating multiple precipitation events into a single composite sample of fixed volume, the variability associated with “first-flush” effects is smoothed, providing a more robust estimation of the average atmospheric deposition flux over the monitored period. Due to spatial and temporal differences in precipitation frequency and intensity during the monitoring period, particularly during the heating season, the number of volume-weighted composite samples collected at each site was not identical (Kikinda, *n* = 15; Sombor, *n* = 13; Sremska Mitrovica, *n* = 12). These differences reflect local rainfall patterns rather than differences in sampling effort, as the same sampling protocol and collection criteria (1 L cumulative precipitation volume) were applied at all locations.

### 2.3. Analytical Methods

Organic micropollutants were determined using gas chromatography–mass spectrometry (GC-MS, Agilent 7890B coupled with 5977B MSD) equipped with an HP-5MS capillary column. Rainwater samples (1 L) were subjected to liquid–liquid extraction using dichloromethane (3 × 20 mL). The combined extracts were dried over anhydrous sodium sulphate and concentrated to 1 mL under a gentle nitrogen stream. By reducing the sample volume from 1000 mL to 1 mL, the analytical window was significantly extended, ensuring that even minor washout events were quantifiable within the linear range of the GC-MS system. Sixteen priority PAHs [9], six priority phthalates (PAE) and BTEX (Table 1) were quantified using external calibration with certified standards. Analysis was performed using a methodology described in the work of Kalkan et al., 2025, Kartalovic et al., 2022, and Habschied et al., 2023 [10,11,12]. Briefly, the analysis was performed under the following conditions. The instrument conditions for BTEX analysis included an injection temperature of 250 °C and an MSD transfer line temperature of 280 °C. The GC oven program started at an initial temperature of 40 °C (held for 3 min), ramped up to 150 °C at 20 °C min^−1^, and was finally held for 1.5 min. Injection was performed in splitless mode with an injection volume of 1 µL. The mass spectrometer was operated in selected ion monitoring (SIM) mode, tracking specific quantifier and qualifier ions for each target compound. For PAE analysis, the injection and MSD transfer line temperatures were both set to 280 °C. The oven program was as follows: initial temperature of 90 °C (held for 1 min), increased to 210 °C at 15 °C min^−1^ (held for 2 min), then at a rate of 5 °C min^−1^ to 240 °C (held for 5 min), followed by an increase of 5 °C min^−1^ to 250 °C, and a final increase of 25 °C min^−1^ to 300 °C (held for 4 min). The column temperature program for PAH determination was set with an initial temperature of 50 °C for 24 s, followed by a gradient of 25 °C min^−1^ to 195 °C (held for 90 s), then a gradient of 8 °C min^−1^ from 195 °C to 265 °C, and a final ramp of 20 °C min^−1^ to 315 °C, which was maintained for 75 s. The detector temperature during PAH analysis was kept at 280 °C.

The QA/QC measures were implemented throughout the sampling campaign and analytical procedure. Stainless-steel funnels and amber glass bottles were used to minimize contamination from plastic materials, particularly for phthalate analysis. All sampling equipment was thoroughly cleaned before deployment. Samples were transported under cooled conditions and stored at 4 °C prior to extraction and analysis. Limits of detection ranged from 0.01 to 0.05 µg L^−1^. Method recoveries were between 85% and 105%, with relative standard deviations below 10%. Quality control included procedural blanks, duplicate samples, and calibration verification standards to evaluate analytical performance and potential contamination.

### 2.4. Data Analysis

Statistical processing and visualization were conducted in R [13]. Prior to hypothesis testing, the distribution of each variable was assessed using normality test (Shapiro–Wilk). Analytes with concentrations below the limit of quantification (LOQ) were treated as censored data; for descriptive statistics, these were replaced with LOQ/2, while in tables they are reported as <LOQ. Homogeneity of variances was evaluated using the Brown-Forsythe test. Depending on the distributional properties of the data, differences among sampling locations were evaluated using either parametric or non-parametric approaches. For normally distributed data with homogeneous variances, one-way analysis of variance (ANOVA) was applied, followed by Tukey’s *post-hoc* test for multiple comparisons. In cases where assumptions of normality or homoscedasticity were not met, the non-parametric Kruskal–Wallis test was used, followed by Dunn’s multiple comparisons test. To assess spatial variability, analyses were conducted for both individual compounds and grouped variables. ΣPAE concentrations met assumptions of normality and homogeneity of variance and are therefore presented as mean ± *SD*, while ΣPAH and ΣBTEX concentrations showed non-normal distributions and are presented as median (min–max). In addition to total concentrations (ΣPAH, ΣBTEX, ΣPAE), PAHs were further classified into low molecular weight (LMW; 2–3 rings), medium molecular weight (MMW; 4 rings), and high molecular weight (HMW; 5–6 rings) fractions. Given the high number of individual analytes, statistical results for individual compounds are primarily presented in the Supplementary Information (SI), while the main text focuses on total concentrations and grouped variables to provide a more robust and interpretable assessment of spatial patterns. All statistical tests were two-tailed, and differences were considered statistically significant at *p* < 0.05.

Source identification of organic micropollutants in rainwater was performed using a suite of widely accepted diagnostic ratios to differentiate between petrogenic and pyrogenic emission sources. For PAHs, the ratios Fla/(Fla + Pyr), Ant/(Ant + Phe), and LMW/HMW were calculated. Specifically, the Fla/(Fla + Pyr) ratio was used to distinguish between petroleum-related inputs (<0.4) and combustion-derived sources (>0.5), while Ant/(Ant + Phe) values exceeding 0.1 were indicative of pyrogenic origins [14,15]. Furthermore, the LMW/HMW ratio (low-molecular-weight vs. high-molecular-weight PAHs) provided additional insight into the relative contribution of petrogenic vs. pyrogenic inputs [1]. It should be noted that PAH diagnostic ratios may exhibit reduced reliability in environments affected by mixed emission sources and atmospheric transformation processes. Weathering, long-range transport, and source overlap can alter the original PAH composition and thereby influence ratio-based source assignments. Therefore, diagnostic ratios were interpreted as supporting indicators and were complemented by multivariate statistical analyses (PCA and correlation analysis) to improve source apportionment reliability [15].

Regarding BTEX, the B/T (benzene/toluene) ratio was used as an indicator of traffic-related versus industrial emissions, whereas the T/X (toluene/xylene) ratio was used to assess the photochemical age of air masses and the degree of atmospheric degradation [7].

To explore the underlying relationships between pollutants and sampling sites, multivariate statistical techniques were employed. Spearman rank correlation was used to evaluate monotonic relationships between different pollutant groups. To reduce data dimensionality and identify dominant emission patterns, Principal Component Analysis (PCA) was performed. Prior to PCA, all data were auto-scaled (mean-centred and scaled to unit variance) to eliminate the influence of different measurement units and ensure that each variable contributed equally to the model. Multivariate analysis and data ordination were implemented using specialised R packages, including FactoMineR for PCA execution and vegan for environmental data statistics [16,17]. This approach ensured a robust, reproducible, and standardised interpretation of the pollutant transformation pathways.

### 2.5. Estimated Annual Atmospheric Deposition Fluxes

The deposition (atmospheric) flux $F_{a}$ was calculated as the product of the mean rainwater concentration C and the annual rainfall depth R:

$$F_{a}=C\times R$$

where F is the annual flux (in µg m^−2^ yr^−1^), C is the mean concentration (in µg L^−1^) derived from the bulk rainwater samples, and R is the annual precipitation sum (in mm, equivalent to L m^−2^) [18,19].

For the Vojvodina region, long-term climatological data indicate that the average annual precipitation ranges from about 550 to 650 mm, with slightly lower values in the northeastern part (Kikinda) and higher values in western-central locations such as Sombor and Sremska Mitrovica [20]. In this study, annual rainfall sums of 560 mm, 610 mm, and 630 mm were adopted for Kikinda, Sombor, and Sremska Mitrovica, respectively, in line with historical precipitation statistics for these stations [20,21].

Using these values R and the mean concentrations, the annual deposition fluxes of ∑PAH, ∑BTEX, and ∑PAE were estimated for each site. The variability of the calculated fluxes was expressed through standard deviation (*SD*), derived from the variance of the measured concentrations (*SD*_flux_ = *SD*_conc_ × R). This approach provides a first-order assessment of the atmospheric burden delivered to the soil and surface-water systems and enables comparison with literature values of PAH and PAE deposition fluxes in other urban and industrial regions of Europe [19,22].

### 2.6. Health Risk Assessment

The potential human health risks associated with exposure to organic micropollutants (BTEX and PAHs) in rainwater via the ingestion pathway were assessed using standard methodologies recommended by the United States Environmental Protection Agency (USEPA).

The Chronic Daily Intake (CDI, mg kg^−1^ day^−1^) for the ingestion pathway was calculated using the following equation:

CDI = C × IR × EF × ED/BW × AT

where C is the concentration of the target pollutant in rainwater (mg L^−1^), and the remaining exposure parameters are defined in Table 2.

Carcinogenic risk associated with PAHs was evaluated through the calculation of the Incremental Lifetime Cancer Risk (ILCR), defined as:

ILCR = CDI × CSF

where CDI represents the chronic daily intake, and CSF is the cancer slope factor. This approach enables quantification of the probability of developing cancer over a lifetime due to exposure to carcinogenic compounds [23].

Non-carcinogenic risk was assessed using the Hazard Quotient (HQ), calculated as:

HQ = CDI/R_f_D

where RfD denotes the substance-specific reference dose mg kg^−1^ day^−1^. HQ values below 1 indicate negligible non-carcinogenic risk, whereas values above 1 suggest potential adverse health effects.

All exposure parameters, including ingestion rates, body weight, and exposure duration, were derived from standardised values provided in the Exposure Factors Handbook [23], ensuring consistency with international risk assessment frameworks.

**Table 2:** Standardised exposure parameters used for CDI, ILCR, and HQ calculations.

Parameter	Symbol	Adults	Children	Unit	Reference
Ingestion rate	IR	2.0	1.0	L day^−1^	USEPA [24]
Body weight	BW	70	15	kg	USEPA [24]
Exposure frequency	EF	350	350	days year^−1^	USEPA [24]
Exposure duration	ED	30	6	years	USEPA [24]
Averaging time (carcinogenic)	AT	25,550	25,550	days	USEPA [24]
Averaging time (non-carcinogenic)	AT	10,950	2190	days	USEPA [24]
Cancer slope factor (for BaP)	CSF	7.3	7.3	(mg kg^−1^ day^−1^)^−1^	USEPA IRIS

Standard exposure parameters for adults and children were applied in accordance with the USEPA Exposure Factors Handbook to ensure age-specific cumulative exposure assessment. These parameters allow differentiation of cumulative exposure between sensitive (children) and adult populations.

## 3. Results & Discussion

### 3.1. Spatial Variability

The analysis of physicochemical parameters and total concentrations of organic pollutants in rainwater samples across Vojvodina indicates significant spatial variability, as shown in Table 3. These results demonstrate a strong east–west pollution gradient driven by industrial activity and local emission sources. The highest ΣBTEX and ΣPAE concentrations were recorded in Kikinda, where the total concentrations of ∑BTEX and ∑PAE exceeded the levels measured in Sombor and Sremska Mitrovica. This gradient directly reflects the intense industrial and petrochemical pressure in the North Banat District. While the pH of the rainwater remained relatively neutral, the high electrical conductivity observed in Sremska Mitrovica suggests a complex atmospheric burden of inorganic ions accompanying the organic micropollutants.

Based on the mean concentrations in Table 3 and the assigned annual rainfall values (560 mm for Kikinda, 610 mm for Sombor, and 630 mm for Sremska Mitrovica), the calculated estimated annual atmospheric deposition fluxes of ∑PAH, ∑BTEX, and ∑PAE are presented in Table 4. The highest fluxes were observed in Kikinda, reflecting the combined influence of industrial emissions and moderate precipitation. Lower fluxes in Sombor and Sremska Mitrovica indicate a contribution of atmospheric washout to the overall pollutant load in these urban-residential environments.

*BTEX compounds*

**Figure 2:**
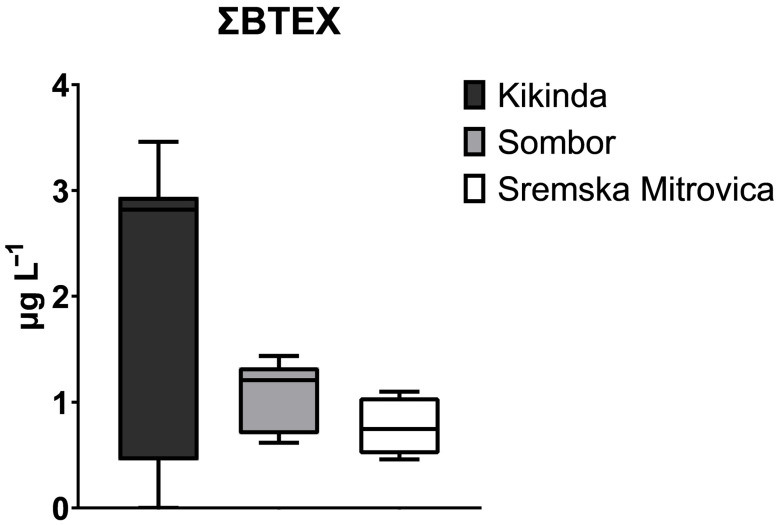
Median (min–max) concentrations (µg L^−1^) of ΣBTEX in rainwater samples from Kikinda, Sombor and Sremska Mitrovica.

No significant spatial differences were observed for sum BTEX compounds (Figure 2), whereas the concentrations of individual BTEX compounds significantly differed among the sampling locations (Figure S1). Toluene concentrations were highest at the traffic-residential site (Sremska Mitrovica), followed by the urban background site (Sombor), while the lowest levels were recorded at the industrial site (Kikinda). These differences were statistically significant between all site pairs (*p* < 0.01). In contrast, ethylbenzene and both xylene isomers (*o,p*-xylene and *m*-xylene) showed the highest concentrations at the industrial site (Kikinda), with significantly lower levels at Sombor and Sremska Mitrovica (*p* < 0.01). The differences between Kikinda and the other two locations were particularly pronounced for *o,p*-xylene, where concentrations were an order of magnitude higher. No consistent significant differences were observed between Sombor and Sremska Mitrovica for ethylbenzene and xylene isomers.

BTEX profiles were dominated by toluene and xylene, indicating industrial emissions rather than traffic sources. Despite the significant enrichment factor (1000:1) achieved through sample preconcentration, benzene remained consistently below the method quantification limit (>MQL). This absence, coupled with elevated levels of toluene and xylenes, reinforces the conclusion that the detected VOCs originate from industrial solvent usage and petrochemical processes rather than mobile combustion sources. Atmospheric scavenging of BTEX showed a distinct industrial signature across all sites, with benzene concentrations below the detection limit. Following the methodology established by Kalkan et al. [12], the B/T near zero definitively excludes road traffic as the primary source and points to non-combustion industrial emissions such as solvent evaporation and petrochemical processing [7]. Additionally, the T/X > 1 indicates fresh emissions from nearby industrial sources rather than aged air masses. As emphasised by Kalkan et al. [12], BTEX washout efficiency depends directly on local emission inventories; in Kikinda, elevated toluene levels (Figure S1) result from proximity to oil fields and refinery operations. Differences in Henry’s law constant contribute to the preferential scavenging of specific BTEXs. The dominance of toluene and xylenes over benzene observed in Kikinda is consistent with emission profiles found in other petrochemical clusters globally, where fugitive emissions from storage tanks prevail over combustion [24,25].

*Phthalate compounds*

**Figure 3:**
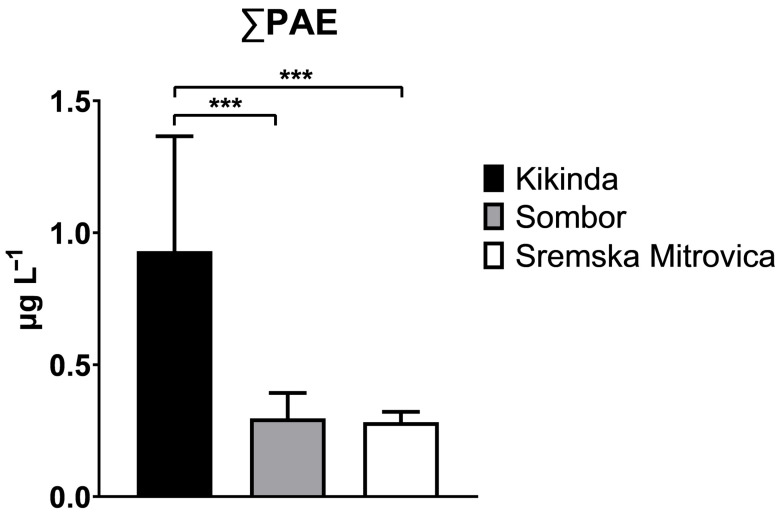
Mean (±*SD*) concentrations (µg L^−1^) of ΣPAE in rainwater samples from Kikinda, Sombor and Sremska Mitrovica. Significant differences are noted with *** (*p* < 0.001; Brown-Forsythe ANOVA followed by *post-hoc* Dunnett’s T3 multiple comparisons test).

Total ΣPAE concentrations exhibited clear spatial variability among the sampling locations (Figure 3), with the highest levels observed at the industrial site (Kikinda), followed by Sombor and Sremska Mitrovica. The differences between Kikinda and the other two sites were statistically significant (*p* < 0.05), while no consistent significant differences were observed between Sombor and Sremska Mitrovica. Analysis of individual phthalate compounds revealed consistent patterns across sites (Figure S2). DMP concentrations were significantly higher at Kikinda compared to both Sombor and Sremska Mitrovica (*p* < 0.05), with minor but significant differences also observed between the two non-industrial sites. A similar trend was observed for DEP, which showed significantly elevated concentrations at Kikinda relative to the other locations (*p* < 0.001). DiBP and DBP exhibited the most pronounced spatial differences, with concentrations at Kikinda significantly exceeding those at both Sombor and Sremska Mitrovica (*p* < 0.001). In contrast, differences between Sombor and Sremska Mitrovica were not statistically significant for these compounds. DEHP followed the same general pattern, with significantly higher concentrations at Kikinda compared to the other sites (*p* < 0.01), while no significant differences were observed between Sombor and Sremska Mitrovica.

Phthalate esters, dominated by DEHP (>70%), were ubiquitous across all samples. The concentration measured in Kikinda was high relative to values typically reported for European urban rainwater (typically 0.1–2.0 μg L^−1^) [3]. Even in Sombor, levels suggest PAEs have become an integral component of the regional hydrological cycle. These compounds, originating from plastic material degradation and industrial additives, readily adsorb onto fine particles efficiently scavenged by precipitation [26]. The widespread DEHP presence indicates a strong link between atmospheric microplastic degradation and phthalate release, highlighting plastic pollution as a secondary source of airborne pollutants. The ubiquitous presence of DEHP in Vojvodina’s rainwater suggests that these compounds have reached a ‘steady state’ in the regional troposphere, similar to observations in major European metropolitan areas [3].

*PAH Compounds*

**Figure 4:**
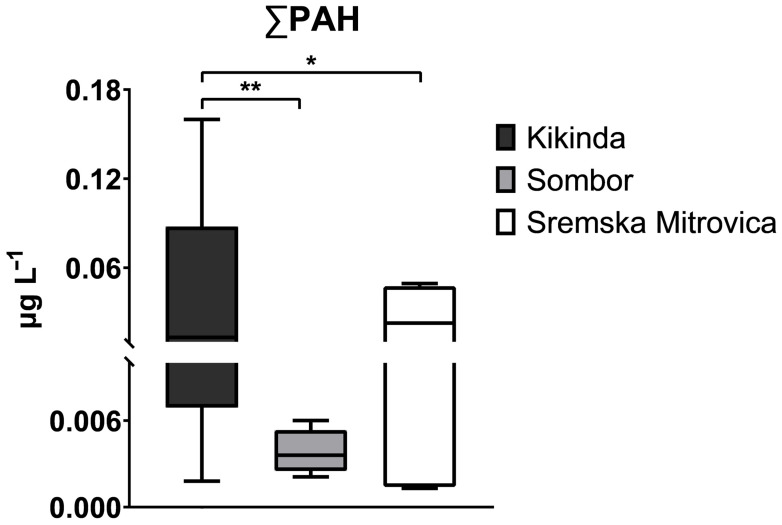
Median (min–max) concentrations (µg L^−1^) of ΣPAH in rainwater samples from Kikinda, Sombor and Sremska Mitrovica. Significant differences are noted with * (*p* < 0.05) and ** (*p* < 0.01; Kruskal–Wallis followed by *post-hoc* Dunn’s multiple comparisons test).

Total ΣPAH showed spatial variability among the sampling locations (Figure 4), with higher values observed at the industrial site (Kikinda) compared to Sombor and Sremska Mitrovica (*p* = 0.003; *p* = 0.04, respectively). When PAHs were grouped according to molecular weight, ΣLMW exhibited the most pronounced spatial pattern. Concentrations were highest at Kikinda, followed by Sremska Mitrovica and Sombor, with a statistically significant difference observed between Kikinda and Sombor (*p* < 0.05; Figure S3). No significant differences were detected between other sites. In contrast, ΣMMW did not show statistically significant differences among the sampling locations, despite slightly elevated concentrations at Sremska Mitrovica. Similarly, ΣHMW exhibited comparable concentrations across all sites, with no significant spatial variation detected.

LMW PAHs, primarily Nap and Phe, dominated this distribution, reflecting precipitation scavenging selectivity. Gas-phase LMW PAHs are more efficiently removed than HMW isomers bound to particulate matter [1]. The enrichment of LMW PAHs in rainwater, as seen in our results, confirms the ‘scavenging ratio’ theory where gas-phase compounds are more readily absorbed into falling droplets compared to soot-bound heavy PAHs [8].

**Figure 5:**
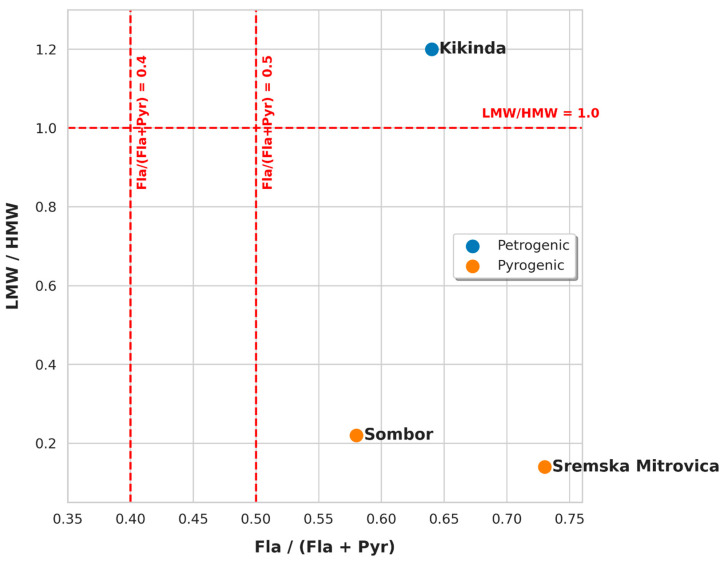
Diagnostic ratio plot of Fla/ (Fla + Pyr) versus LMW/HMW. Dashed reference lines indicate commonly used threshold values for distinguishing petrogenic and pyrogenic PAH sources ((Fla/(Fla + Pyr) = 0.4 and 0.5; LMW/HMW = 1.0).

Regarding the diagnostic ratios, in Kikinda, an LMW/HMW ratio of 1.20 indicates petrogenic (petroleum) origins from petrochemical industry influence (Figure 5). Conversely, Sremska Mitrovica and Sombor exhibit strong pyrogenic signatures (Fla/(Fla + Pyr) > 0.5), characteristic of biomass combustion and residential heating during winter [14]. These findings align with Kalkan et al. [12], who identify meteorological conditions and seasonal heating practices as primary drivers of organic pollutant profiles in the Pannonian Basin urban environments.

### 3.2. Integrated Source Interpretation and Regional Comparison

**Figure 6:**
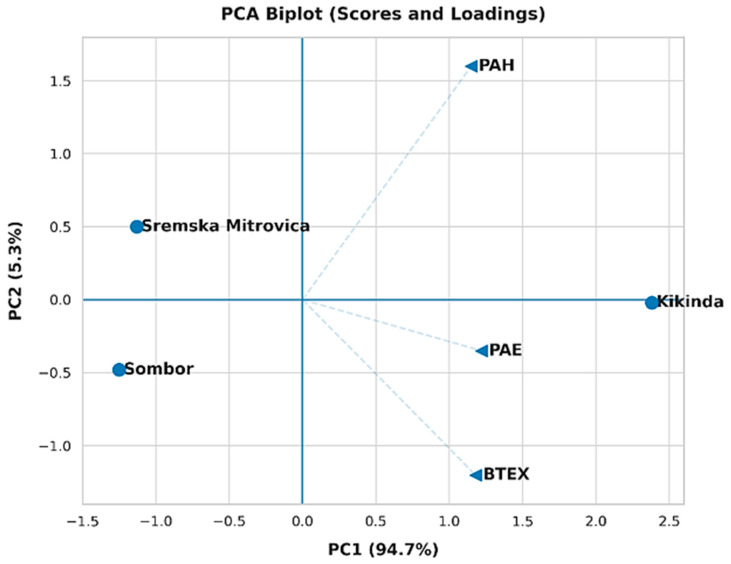
PCA biplot (scores and loadings) showing the distribution of sampling sites and the contribution of PAHs, PAEs, and BTEX.

The chemical fingerprinting of rainwater samples reveals a pronounced spatial contrast across Vojvodina, primarily driven by local industrial activities and seasonal heating practices. This differentiation is clearly reflected in the distribution of diagnostic ratios and multivariate analysis (Figure 5 and Figure 6).

Kikinda exhibits a dominant petrogenic signature, as evidenced by the LMW/HMW ratio exceeding unity (1.20, Figure 5). In environmental forensics, such values are indicative of petroleum-derived inputs, reflecting the presence of fresh, uncombusted hydrocarbons. Unlike locations where HMW PAHs dominate due to combustion-related soot formation, the rainwater in Kikinda is enriched in LMW compounds. This pattern suggests efficient scavenging of gas-phase hydrocarbons originating from nearby oil and gas extraction fields and refinery operations in the Northern Banat region.

This interpretation is further supported by the diagnostic ratio plot (Figure 5), where Kikinda clearly clusters within the petrogenic domain. Additionally, the PCA biplot (Figure 6) indicates a partial separation of Kikinda from the other sites, consistent with its higher contribution of volatile and industrially associated compounds. Nevertheless, because PC2 explained only 5.3% of the total variance, the observed separation should be interpreted with caution and in conjunction with diagnostic ratios and correlation analysis. Correlation analysis further supported compound-specific relationships among organic pollutants (Figure S4). Significant BTEX–PAE correlations were observed mainly between alkylated BTEX compounds and selected phthalates. Ethylbenzene was positively correlated with DMP, DiBP, BBP, and DEHP (*r* = 0.35–0.52, *p* < 0.05), while *o,p*-xylene was positively correlated with BBP and DEHP (*r* = 0.33–0.36, *p* < 0.05). Similarly, *m*-xylene showed significant positive correlations with DMP, DiBP, BBP, and DEHP (*r* = 0.34–0.54, *p* < 0.05). In contrast, toluene showed significant negative correlations with DMP, DEP, DiBP, DBP, and DEHP (*r* = from −0.42 to −0.74, *p* < 0.01). These results suggest partial source overlap between selected BTEX compounds and PAEs, particularly for ethylbenzene/xylene isomers and phthalates, whereas the inverse correlations with toluene indicate that BTEX–PAE relationships were not uniform. In contrast, Sremska Mitrovica and Sombor display a clear pyrogenic signature, characterized by elevated Fla/(Fla + Pyr) ratios (>0.5, Figure 5). The value of 0.73 observed in Sremska Mitrovica is particularly indicative of low-temperature biomass and coal combustion. Considering that the sampling campaign was conducted during the heating season, these results strongly suggest that residential heating systems represent a major source of atmospheric PAHs in these areas. The predominance of HMW PAHs (>80%) further supports this interpretation, as these compounds are typically associated with particulate matter generated during incomplete combustion processes.

The spatial trends are consistent with these findings, showing lower total concentrations in Sombor and Sremska Mitrovica compared to Kikinda, but a higher relative contribution of combustion-derived compounds. This pattern is further reinforced in PAHs, exhibiting a distinct distribution compared to BTEX and PAEs.

When compared with previous studies, the PAH concentrations observed in this work fall within the range reported for industrial and urban environments across Europe and Asia. The levels detected in Sremska Mitrovica and Sombor are consistent with urban background conditions in the Balkan region, where wintertime atmospheric profiles are largely governed by biomass burning [27].

A notable distinction of this study is the identification of a strong petrogenic signal in Kikinda, which contrasts with findings from major European urban centres such as Paris or Belgrade, where PAH profiles are typically dominated by pyrogenic sources related to traffic and heating, resulting in lower LMW/HMW ratios [28,29].

In addition to PAHs, PAE concentrations further support the influence of industrial and urban activities. The levels detected in Kikinda fall within the range reported for European urban rainwater (0.1–2.0 μg L^−1^) [30], suggesting that plastic degradation and industrial additives contribute to atmospheric PAE loading. Their consistent presence across all sites indicates that phthalates have become a ubiquitous component of the regional atmospheric cycle.

Overall, the combined analysis of diagnostic ratios, spatial distribution, and multivariate statistics demonstrates that atmospheric washout processes in Northern Serbia are governed by a complex interplay of industrial emissions, residential heating, and secondary atmospheric transformations. Future research should quantify the long-term accumulation of these contaminants in soil and surface waters using the calculated atmospheric deposition fluxes, which would directly link rainwater quality to the agricultural and ecosystem risks in the Vojvodina region. Furthermore, it is important to acknowledge that the present study is temporally confined to a single heating season (December 2025–April 2026). While wet deposition dynamics during this winter-spring period are highly pronounced due to intensive residential heating and stable atmospheric conditions, the absence of a warm-season comparator represents a limitation. Consequently, the calculated annual deposition fluxes should be interpreted as first-order estimates rather than definitive annual cycles. Future multi-season monitoring is warranted to fully capture the seasonal variability, particularly the potential shifts in PAH and PAE profiles driven by photochemical degradation and different emission patterns during the summer months.

### 3.3. Health Risk Assessment

The presence of organic micropollutants in rainwater may pose a potential risk to human health through complex exposure pathways. In addition to direct contact during household or agricultural activities, atmospheric deposition facilitates the infiltration of pollutants into groundwater and their subsequent bioaccumulation within the food chain. To quantify these risks, the toxicological significance of the detected compounds was evaluated through the Incremental Lifetime Cancer Risk (ILCR) for PAHs and the Hazard Quotient (HQ) for the non-carcinogenic systemic toxicity of BTX compounds, with results summarised in Table 5. The health risk assessment was performed considering the cumulative exposure from all analysed samples at each site. The inclusion of standard deviations for ILCR and HQ values accounts for the environmental variability of pollutant concentrations, providing a more comprehensive evaluation of the potential health impact on both adult and paediatric populations.

#### 3.3.1. Carcinogenic Risk of PAHs

The carcinogenic risk associated with PAHs was determined by integrating three primary exposure routes: oral ingestion, dermal absorption, and the inhalation of volatilized compounds or aerosols. As shown in Table 4, the calculated ILCR values exhibited a pronounced spatial gradient. The values for Kikinda (8.9 × 10^−6^ for adults) were significantly higher than those recorded in Sombor and Sremska Mitrovica, aligning with the “acceptable or tolerable risk” range 10^−6^ to 10^−4^ defined by the US EPA [31]. However, the risk for children in Kikinda (2.1 × 10^−5^ approached the precautionary limit, which is directly attributable to the high concentrations of BaP. This concentration markedly exceeds the maximum permissible level (0.010 µg L^−1^) stipulated by both European Union and Serbian national legislation [20,32,33]. These findings suggest that atmospheric washout serves as a critical non-point source of high-TEF (Toxic Equivalency Factor) compounds, potentially compromising the regional hydrosphere [34].

#### 3.3.2. Non-Carcinogenic Risk of BTEX

The non-carcinogenic risk was assessed using the Hazard Quotient, comparing the estimated daily intake against Reference Dose (RfD) values obtained from the EPA Integrated Risk Information System. Despite the elevated concentrations of toluene and xylenes observed in Kikinda, the resulting HQ values remained well below the unity threshold (HQ < 1.0), reaching a maximum of 6.9 × 10^−4^. Such results indicate that BTX levels in the rainwater of Northern Serbia do not present an immediate systemic health threat via wet deposition. This is consistent with observations by Šostaric et al. [7], who noted that the high volatility and relatively high RfD values of BTEX compounds often result in lower toxicological risks through water-based pathways compared to direct atmospheric inhalation.

#### 3.3.3. Demographic Vulnerability and Exposure Pathways

A significant outcome of this assessment is the heightened vulnerability of the paediatric population, whose ILCR values in Kikinda were more than double those of the adult group. This disparity is fundamentally linked to physiological and behavioural factors, including lower average body weight and higher relative intake rates per unit of body mass [23]. Furthermore, children often experience higher exposure through dermal contact and accidental ingestion, which are pathways frequently overlooked in standard risk assessments [35,36]. The findings underscore the necessity of developing integrated monitoring frameworks that prioritise sensitive demographic subgroups in industrialized regions of the Pannonian Basin.

## 4. Conclusions

The main novelty of this study lies in the integrated assessment of PAHs, PAEs, and BTEXs in rainwater, combined with source apportionment, atmospheric deposition flux estimation, and human health risk assessment across contrasting emission environments in Northern Serbia. The highest ΣBTEX and ΣPAE concentrations were recorded in Kikinda, with a median ΣBTEX of 2.818 µg L^−1^ [< LOQ–3.460] and a mean ΣPAE of 0.930 ± 0.435 µg L^−1^. In contrast, the highest median ΣPAH concentration was observed in Sremska Mitrovica (0.022 µg L^−1^ [0.001–0.049]), followed by Kikinda (0.018 µg L^−1^ [0.001–0.159]) and Sombor (0.003 µg L^−1^ [0.001–0.006]). Estimated annual atmospheric deposition fluxes were highest in Kikinda, with ΣBTEX and ΣPAE reaching 1095 ± 762 and 521 ± 244 µg m^−2^ yr^−1^, respectively, while ΣPAH reached 27.25 ± 31.59 µg m^−2^ yr^−1^. The calculated ILCR values ranged from 8.3 × 10^−8^ to 2.1 × 10^−5^, indicating acceptable but elevated carcinogenic risk in industrial locations, while HQ values remained below 1.0 across all sites. The pronounced petrogenic signature observed in Kikinda highlights the dominant influence of petrochemical activities, while pyrogenic patterns in Sremska Mitrovica and Sombor confirm the role of residential biomass combustion. The ubiquitous presence of PAEs, particularly DEHP, indicates their incorporation into the atmospheric hydrological cycle, likely linked to plastic degradation processes. The dominance of LMW PAHs further confirms the importance of gas-phase scavenging in wet deposition. These findings emphasize the necessity of transitioning from single-compartment monitoring toward integrated atmospheric-hydrological frameworks for accurate environmental risk assessment in industrialized regions of the Pannonian Basin. These results highlight the need for integrating total atmospheric deposition monitoring with soil and water quality assessments. This integration is essential for evaluating the long-term accumulation of pollutants and the associated risks to Vojvodina’s agricultural and aquatic ecosystems.

## Figures and Tables

**Table 1:** Target compounds analysed in the present study and their abbreviations (PAHs, BTEX, and PAEs).

Group	Compound	Abbreviation
Polycyclic aromatic hydrocarbons (PAHs)	Naphthalene	NAP
	Acenaphthylene	ACY
	Acenaphthene	ACE
	Fluorene	FLU
	Phenanthrene	PHE
	Anthracene	ANT
	Fluoranthene	FLA
	Pyrene	PYR
	Benzo[*a*]anthracene	BaA
	Chrysene	CHR
	Benzo[*b*]fluoranthene	BbF
	Benzo[*k*]fluoranthene	BkF
	Benzo[*a*]pyrene	BaP
	Dibenzo[*a,h*]anthracene	DahA
	Benzo[*g,h,i*]perylene	BghiP
	Indeno[1,2,3-*cd*]pyrene	IcdP
Benzene, toluene, ethylbenzene, and xylene isomers (BTEX)	Benzene	BEN
	Toluene	TOL
	Ethylbenzene	EB
	*m,p*-Xylene	*m,p*-XYL
	*o*-Xylene	*o*-XYL
Phthalates (PAEs)	Dimethyl phthalate	DMP
	Diethyl phthalate	DEP
	Di-n-butyl phthalate	DnBP
	Butyl benzyl phthalate	BBP
	Di(2-ethylhexyl) phthalate	DEHP
	Di-n-octyl phthalate	DnOP

**Table 3:** Concentrations of ∑PAH, ∑BTEX and ∑PAE (μg L^−1^) as well as physicochemical parameters, pH and electrical conductivity (EC, µS cm^−1^) in rainwater samples from three locations in Northern Serbia.

Sampling Location	∑PAH (Median, [Min–Max])	∑BTEX (Median, [Min–Max])	∑PAE (Mean ± *SD*)	pH (Mean ± *SD*)	EC (Mean ± *SD*)
**Kikinda** **(*n* = 15)**	0.018 [0.001–0.159]	2.818 [<LOQ–3.460]	0.930 ± 0.435	7.03 ± 0.12	162.48 ± 26.83
**Sombor** **(*n* = 13)**	0.003 [0.001–0.006]	1.208 [0.616–1.437]	0.296 ± 0.09	7.26 ± 0.09	53.47 ± 15.05
**Sremska Mitrovica** **(*n* = 12)**	0.022 [0.001–0.049]	0.747 [0.460–1.099]	0.282 ± 0.03	8.11 ± 0.15	935.99 ± 249.28

**Table 4:** Annual precipitation (R, mm) and mean (±*SD*) corresponding estimated atmospheric deposition fluxes (F*_a_*, µg m^−2^ yr^−1^) calculated from measured concentrations and rainfall at the studied locations of Northern Serbia.

Sampling Location	R	F*_a_*_∑PAH_	F*_a_*_∑BTEX_	F*_a_*_∑PAE_
**Kikinda** (*n* = 15)	560	27.25 ± 31.59	1095 ± 762	521 ± 244
**Sombor** (*n* = 13)	610	2.35 ± 0.82	641 ± 194	181 ± 59
**Sremska Mitrovica** (*n* = 12)	630	15.23 ± 14.75	486 ± 174	178 ± 24

**Table 5:** Estimated Incremental Lifetime Cancer Risk (ILCR) for PAHs and Hazard Quotient (HQ) for BTX compounds across sampling sites.

Sampling Site	∑BaPeq (μg L^−1^)	ILCR Adults	ILCR Children	HQ Toluene (Adults)
**Kikinda** (*n* = 15)	0.0428 ± 0.005	8.9 × 10^−6^ ± 1.1 × 10^−6^	2.1 × 10^−5^ ± 0.3 × 10^−5^	6.9 × 10^−4^ ± 0.8 × 10^−4^
**Sombor** (*n* = 13)	0.0004 ± 0.0001	8.3 × 10^−8^ ± 0.9 × 10^−8^	1.9 × 10^−7^ ± 0.2 × 10^−7^	7.4 × 10^−4^ ± 0.9 × 10^−4^
**Sremska Mitrovica** (*n* = 12)	0.0032 ± 0.0004	6.6 × 10^−7^ ± 0.7 × 10^−7^	1.5 × 10^−6^ ± 0.2 × 10^−6^	7.6 × 10^−4^ ± 1.0 × 10^−4^
**Threshold**	-	10^−6^ to 10^−4^	10^−6^ to 10^−4^	<1.0

## Data Availability

The original contributions presented in this study are included in the article/Supplementary Materials. Further inquiries can be directed to the corresponding author.

## Supplementary Materials

The following supporting information can be downloaded at: https://www.mdpi.com/article/10.3390/jox16030116/s1, Figure S1: Mean concentrations (µg L^−1^) of BTEX compounds in rainwater samples from Kikinda, Sombor, and Sremska Mitrovica. Asterisks indicate statistically significant pairwise differences between sampling sites (** *p* < 0.01, *** *p* < 0.001, **** *p* < 0.0001); Figure S2: Mean *(±SD)* concentrations (µg L^−1^) of PAE compounds in rainwater samples from Kikinda, Sombor, and Sremska Mitrovica. Asterisks indicate statistically significant pairwise differences between sampling sites (* *p* < 0.05, ** *p* < 0.01, **** *p* < 0.0001); Figure S3: Mean (±*SD*) concentrations (µg L^−1^) of PAH compounds in rainwater samples from Kikinda, Sombor, and Sremska Mitrovica. Asterisks indicate statistically significant pairwise differences between sampling sites (* *p* < 0.05); Figure S4: Spearman correlation matrix showing relationships among measured organic pollutants in rainwater samples. Correlation coefficients (*r*) are displayed within each cell. Colour intensity represents the strength and direction of correlations, with red indicating positive and blue indicating negative associations.
