# Supplementary material for: Atmospheric Washout Dynamics of Organic Micropollutants: A Study of PAH, PAE, and BTEX Concentrations in Rainwater Across Northern Serbia
Source: J Xenobiot. 2026 Jun 20;16(3):116. doi: 10.3390/jox16030116 (PMC13301899; doi:10.3390/jox16030116)
Supplement: Supplementary file 1 [file jox-16-00116-s001.zip › jox-4330042-supplementary.pdf]

Supplementary Materials: Atmospheric Washout Dynamics of Organic Micropollutants: A Study of PAH, PAE, and BTEX Concentrations in Rainwater Across Northern Serbia

Brankica Kartalović, Rastko Tomanović, Kristina Habschied, Alma Mikuška, Mirta Sudarić Bogojević, Antonije Žunić and Dora Bjedov

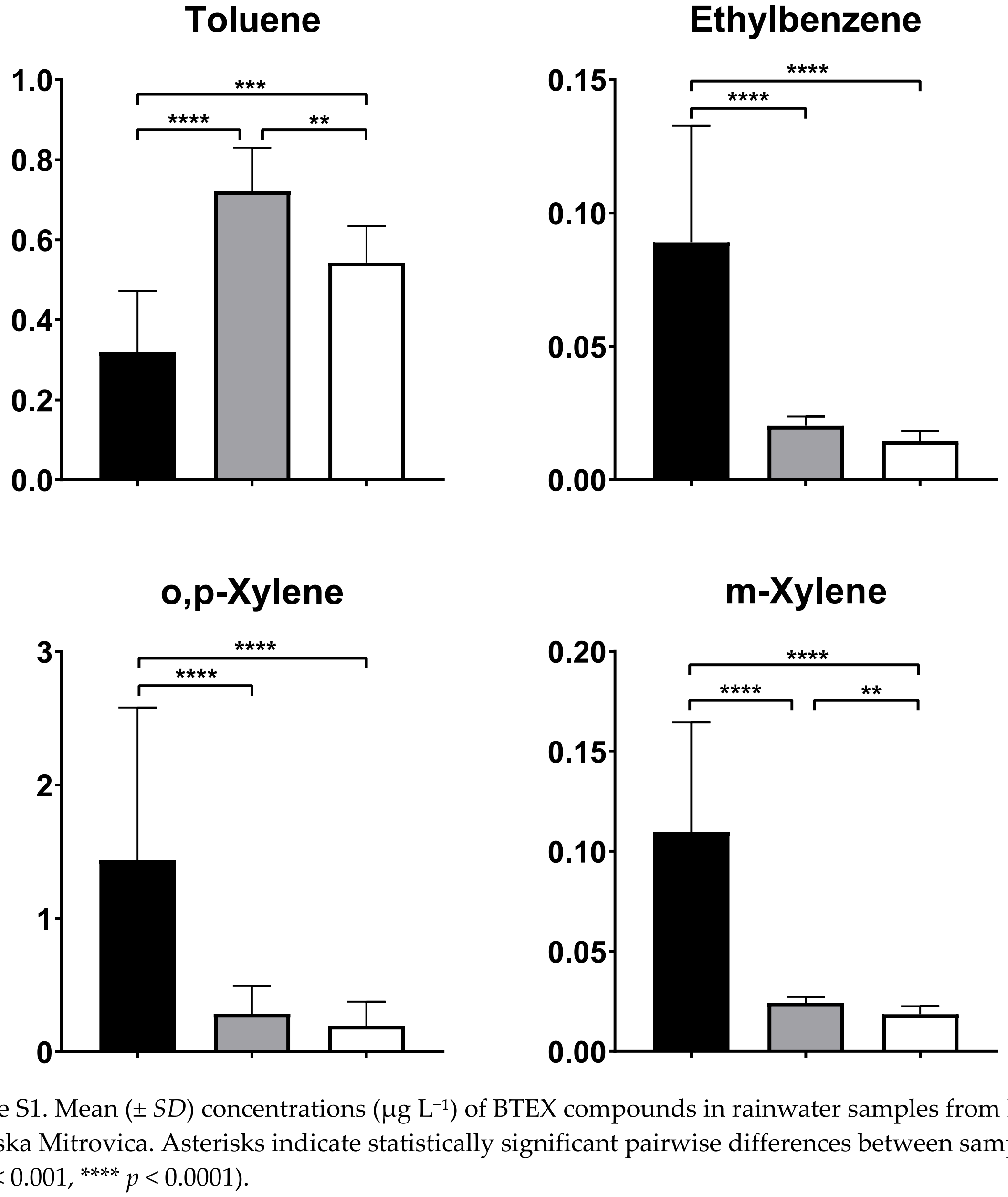

Figure S1. Mean (± *SD*) concentrations (μg L^{-1}) of BTEX compounds in rainwater samples from Kikinda, Sombor, and Sremska Mitrovica. Asterisks indicate statistically significant pairwise differences between sampling sites (** $p < 0.01$, *** $p < 0.001$, **** $p < 0.0001$).

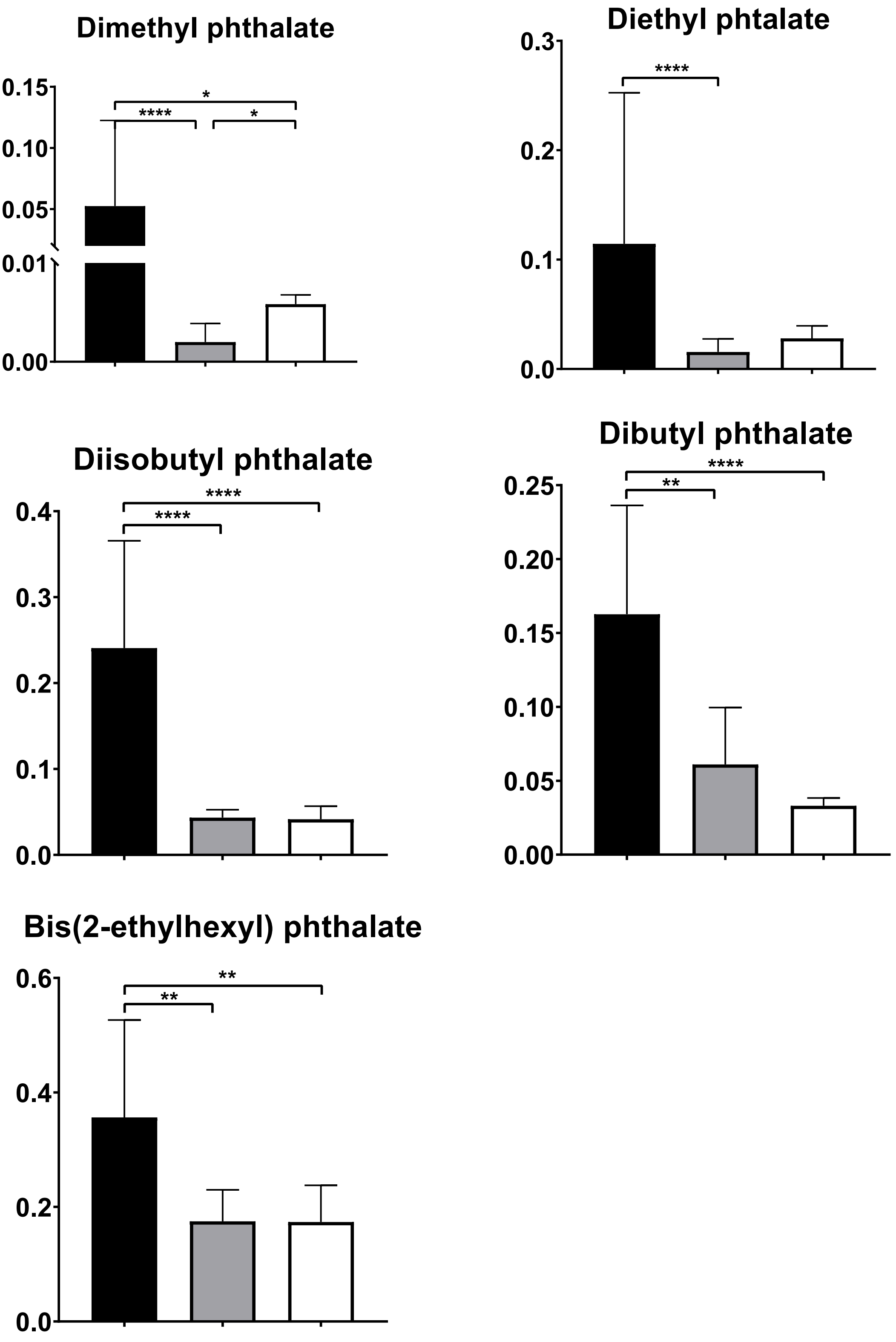

Figure S2. Mean (± *SD*) concentrations (μg L^{-1}) of PAE compounds in rainwater samples from Kikinda, Sombor, and Sremska Mitrovica. Asterisks indicate statistically significant pairwise differences between sampling sites (* $p < 0.05$, ** $p < 0.01$, **** $p < 0.0001$).

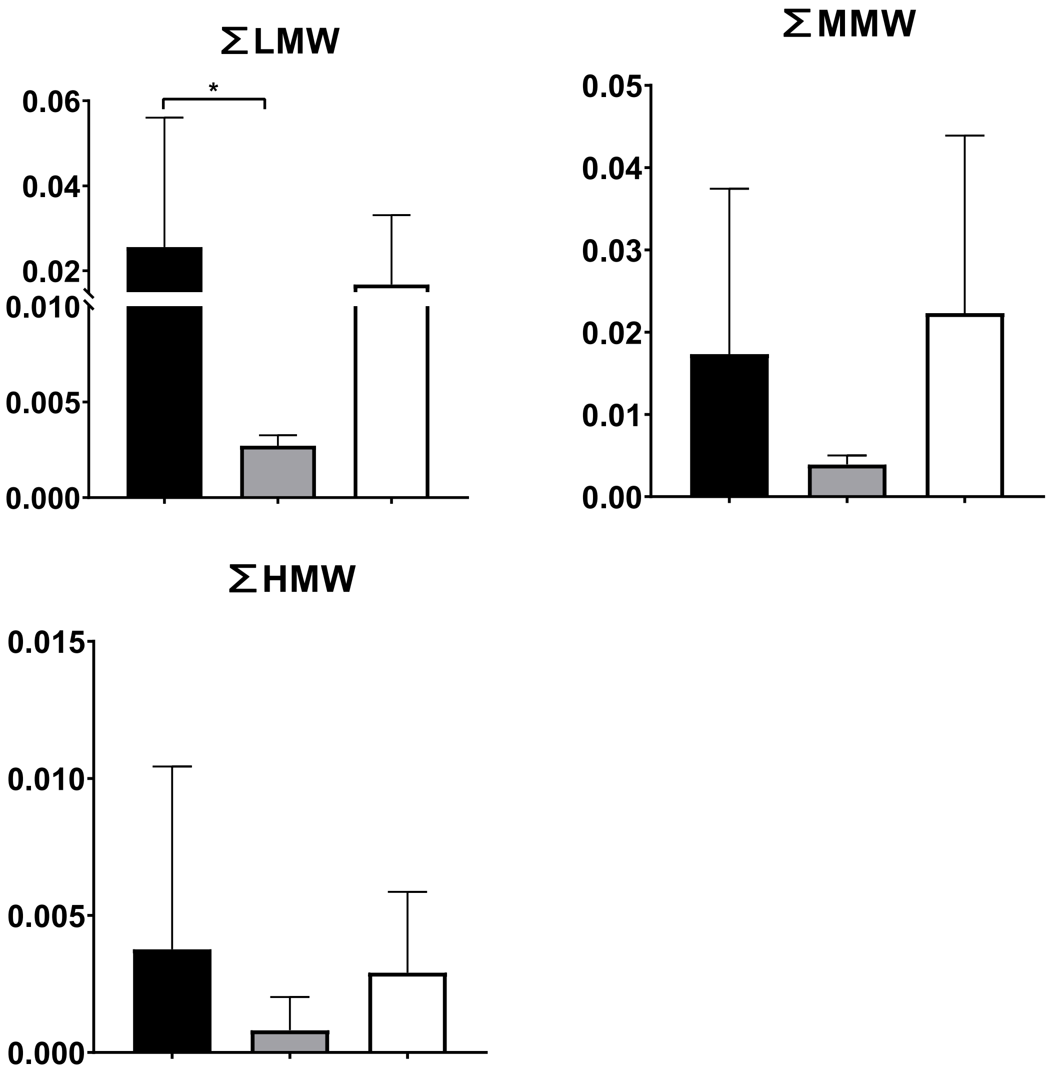

Figure S3. Mean (± *SD*) concentrations (μg L^{-1}) of PAH compounds in rainwater samples from Kikinda, Sombor, and Sremska Mitrovica. Asterisks indicate statistically significant pairwise differences between sampling sites (* $p < 0.05$).

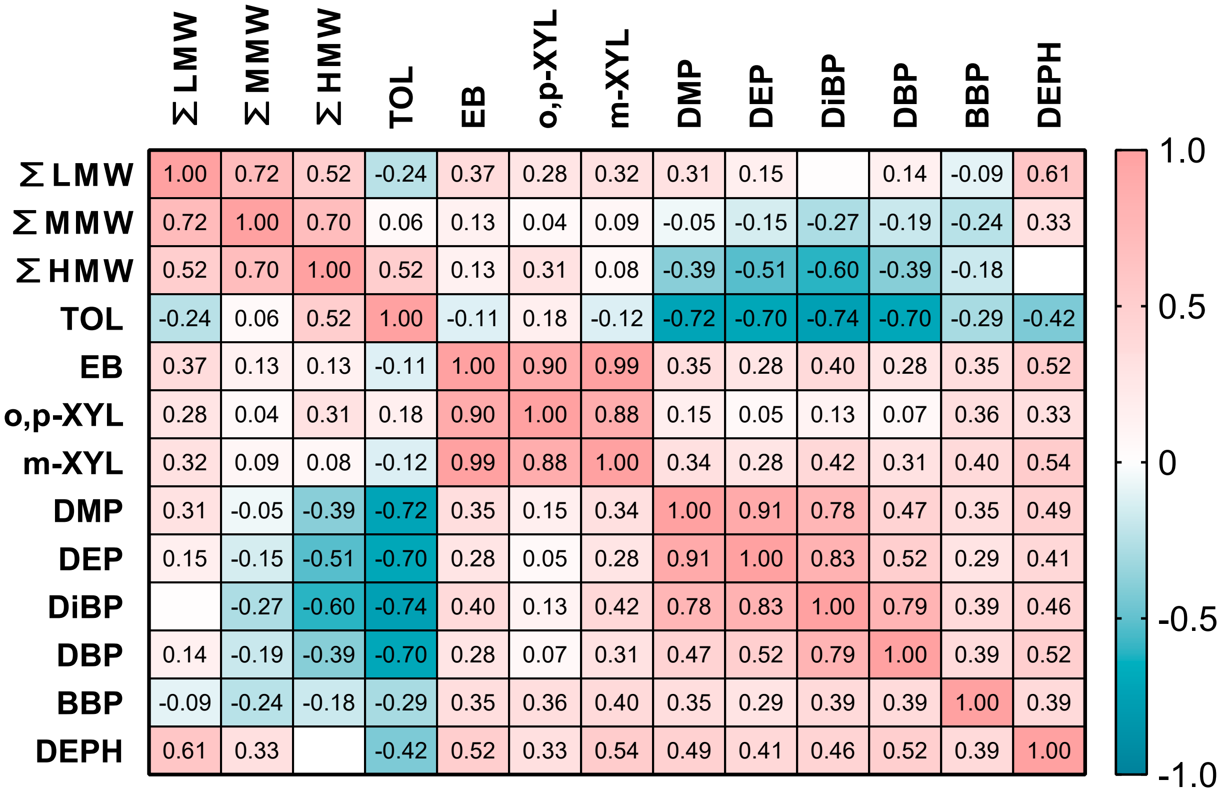

Figure S4. Spearman correlation matrix showing relationships among measured organic pollutants in rainwater samples. Correlation coefficients (*r*) are displayed within each cell. Colour intensity represents the strength and direction of correlations, with red indicating positive and blue indicating negative associations.
